# MERTK is a host factor that promotes classical swine fever virus entry and antagonizes innate immune response in PK-15 cells

**DOI:** 10.1080/22221751.2020.1738278

**Published:** 2020-03-14

**Authors:** Guanglai Zheng, Lian-Feng Li, Yuexiu Zhang, Liang Qu, Wenjing Wang, Miao Li, Shaoxiong Yu, Mo Zhou, Yuzi Luo, Yuan Sun, Muhammad Munir, Su Li, Hua-Ji Qiu

**Affiliations:** a State Key Laboratory of Veterinary Biotechnology, Harbin Veterinary Research Institute, Chinese Academy of Agricultural Sciences, Harbin, People’s Republic of China; b Division of Biomedical and Life Sciences, Faculty of Health and Medicine, Lancaster University, United Kingdom

**Keywords:** MERTK, TAM receptors, classical swine fever virus, entry, innate immune response

## Abstract

Classical swine fever virus (CSFV) is a member of the genus *Pestivirus* in the *Flaviviridae* family. To date, the host factors required for CSFV entry remain poorly characterized. To identify the functional membrane protein(s) involved in CSFV infection, we analyzed the transcriptomic data from previous studies describing gene expression profiles for CSFV, and found twelve novel candidate proteins. One of these proteins, MERTK, significantly reduced CSFV protein expression by RNA interference screening using a recombinant CSFV that contains a luciferase reporter to measure CSFV protein expression. Furthermore, our results demonstrated that either anti-MERTK antibodies or soluble MERTK ectodomain could reduce CSFV infection in PK-15 cells in a dose-dependent manner. Mechanistically, MERTK interacted with the E2 protein of CSFV and facilitated virus entry. After virus entry, MERTK downregulates of mRNA expression of IFN-β and promotes CSFV infection. Interestingly, the soluble MERTK ectodomain could also reduce the infection of bovine viral diarrhea virus (BVDV), another pestivirus. Taken together, our results suggested that MERTK is a CSFV entry factor that synergistically dampens innate immune responses in PK-15 cells and is also involved in BVDV infection.

## Introduction

Classical swine fever virus (CSFV) belongs to the *Pestivirus* genus within the *Flaviviridae* family, a group of enveloped, single-stranded, positive-sense RNA viruses [1, 2]. The CSFV genome RNA encodes four structural proteins (capsid protein, C and three glycoproteins, E^rns^, E1, and E2) and eight nonstructural proteins (N^pro^, p7, NS2, NS3, NS4A, NS4B, NS5A, and NS5B) [3–5].

The glycoprotein E2 forms homodimers and heterodimers with glycoprotein E1 through disulfide bonds, and the formation of heterodimers is critical for pestivirus entry [6–8]. E1 and E2 proteins are considered to be sufficient to mediate CSFV entry [9]. The glycoprotein E^rns^ lacks the membrane anchor and its conformation may play an important role in host tropism [10]. Heparan sulfate (HS) and laminin receptor (LamR) have been identified as attachment receptors for CSFV, which interact with the E^rns^ protein [4,11]. Porcine CD46 has also been reported to serve as an attachment factor for CSFV [6]. To date, only one membrane protein known as annexin 2 has been found to bind with E2 for promoting viral growth [12]. Other membrane protein(s), which interacts with E2 and mediates CSFV entry into host cells, remains to be elucidated.

MERTK is a member of the TAM (TYRO3, AXL, and MERTK) receptor protein tyrosine kinases, which regulate tissue homeostasis, particularly the phagocytic clearance of apoptotic cells and antagonism of innate immune responses [13,14]. Many reports have shown that the AXL and TYRO3 of the TAM receptors could potentiate the infection of various viruses in different pathways [15]. For example, AXL facilitates Zaire Ebolavirus (ZEBOV) entry by enhancing the macropinocytosis pathway [16]. TYRO3 and AXL can mediate the entry of dengue virus (DENV) into host cells via the clathrin-dependent endocytosis pathway [17–19]. Moreover, AXL plays a pivotal role in mediating Zika virus (ZIKV) entry into human skin cells, neural stem cells, and human glial cells [20–22]. Furthermore, VP1 protein of the non-enveloped polyomavirus simian virus 40 (SV40) can directly interact with AXL for promoting viral infection [23]. However, little information is available on the role of MERTK in viral infections.

In the present study, we found that downregulation of MERTK significantly reduced CSFV infection based on siRNA screening. Moreover, our results indicate that the interaction of E2 and MERTK facilitates CSFV entry and the activation of the tyrosine kinase of the MERTK dampens the innate immune response in porcine kidney (PK-15) cells, providing a potential therapeutic target.

## Materials and methods

### Cells and viruses

Porcine kidney (PK-15), Human embryonic kidney (HEK293 T), and Madin-Darby bovine kidney (MDBK) cells were cultured in Dulbecco’s modified Eagle’s medium (DMEM) (Gibco) supplemented with 10% FBS (Gibco).

CSFV Shimen strain (CSFV-SM), rCSFV-Rluc [24], CSFV HLJZZ2014 strain (CSFV-HLJ) [25] and pseudorabies virus (PRV) TJ strain (PRV-TJ) [26] were propagated in PK-15 cells. The bovine viral diarrhea virus (BVDV) Oregon C24 V strain (BVDV-C24 V) was provided by China Institute of Veterinary Drug Control and propagated in MDBK cells.

### Cell viability assay

Cell viability assay was performed using the cell counting kit-8 (CCK-8) (Dojindo) according to the manufacturer’s instructions.

### RNA interference assay

The siRNAs targeting candidate membrane proteins and negative control were synthesized by GenePharma.

To knock down the target genes, PK-15 cells were plated at a density of 2×10^5^ cells per well in 24-well plates. Simultaneously, the cells were transfected with 120 nM siRNAs by using the X-tremeGENE siRNA transfection reagent (Roche) according to the manufacturer’s instructions. After 48 h, the cells were infected with CSFV-SM or rCSFV-Rluc at a multiplicities of infection (MOI) of 0.01. At 48 hpi, the cells or the supernatants were used to detect viral RNA copies, viral titers or *Renilla* luciferase activity.

### Real-time RT–PCR

Genomic RNA copies of CSFV were quantified by real-time RT–PCR (RT-qPCR) as previously described [27].

### Luciferase activity assay

At 48 hpi, the PK-15 cells infected with rCSFV-Rluc were washed twice with phosphate-buffered saline (PBS), and then lysed with passive lysis buffer (Promega) for 30 min at 4°C. The lysate was collected into 1.5-ml tubes and centrifuged for 5 min at 12,000 × *g*, and then the supernatants were added into white 96-well plates and assayed for *Renilla* luciferase activities using the luciferase reporter assay system (Promega). Luminescence was determined by the TD-20/20 luminometer (Turner Designs) according to the manufacturer’s instructions.

### Immunoprecipitation assay

HEK293 T cells were transfected with 2 μg of pMERTK-Myc and pE2-Flag or pE^rns^-Flag in each well of 6-well plates (Corning). At 48 h post transfection (hpt), the cells were washed twice with cold PBS, lysed with NP-40 buffer (Beyotime) containing 1 mM phenylmethylsulfonyl fluoride (Beyotime) for 30 min at 4°C. After being centrifuged at 12,000 × *g* for 5 min at 4°C, the supernatants were incubated with 30 μl of protein G-Agarose (Sigma) to remove the unspecific binding proteins. Next, the supernatants were incubated with 30 μl of ANTI-FLAG® M2 Affinity Gel (Sigma) for 6 h at 4°C. Then the resins were washed three times with NP-40 buffer and boiled with corresponding 5 × SDS-PAGE loading buffer for 8 min at 100°C. The complexes were then analyzed by Western blotting with the indicated antibodies.

### Flow cytometry assay

PK-15 cells were digested with 2 mM EDTA. The cell surface expression of MERTK was analyzed by staining the cells with rabbit anti-MERTK antibodies (catalog no. ab70693, Abcam) or isotype IgG for 1 h at room temperature. Then the cells were incubated with Alexa-488 donkey anti-rabbit IgG (H + L) (catalog no. A21206, Invitrogen) for 30 min. All flow cytometry experiments were carried out using BD Accuri C6 Flow Cytometer (BD Bioscience) and the data were analyzed by the FlowJo software version 10 (TreeStar).

### Blocking assay

To assess whether soluble MERTK^ED^-His could reduce CSFV infection, CSFV-SM (MOI = 0.01) was preincubated with indicated concentrations of soluble MERTK^ED^-His or BSA for 30 min at 37°C, and then infected PK-15 cells for 48 h at 37°C. For the antibody blocking assays, PK-15 cells were preincubated with a various concentration of anti-MERTK or isotype rabbit IgG for 30 min and then were infected with CSFV-SM (MOI = 0.01) in the presence of antibodies for 48 h. Then the viral genome copies and progeny viral titers were detected.

### Surface plasmon resonance (SPR) analysis

The interaction between purified MERTK^ED^-His and E2^ED^-His was assessed by using Biacore T200 instrument (GE Healthcare) at room temperature. The CM5 biosensor chip (GE Healthcare) was immobilized with MERTK^ED^-His according to the manufacturer’s amine-coupling kit protocols (GE Healthcare). Another channel without immobilized ligand was used as a reference to account for non-specific binding to the sensor. Both channels were then blocked with ethanolamine. The soluble E2^ED^-His protein was loaded at concentrations of 0, 0.375, 0.75, 1.5, 3, 6 and 12 μM, and then the binding responses were recorded. All proteins used in the experiment were exchanged into the HBS-EP (10 mM HEPES, pH 7.4, 150 mM NaCl, and 0.05% Tween 20). The *K_D_* value for the interaction was calculated using the Biacore T200 evaluation software (GE Healthcare).

### Virus entry assays

For the virus entry assay, PK-15 cells were washed with PBS twice and then were incubated with CSFV-SM (MOI = 1 or 0.1) for 2 h at 37°C to allow entry, and subsequently washed twice with PBS. To remove surface-bound virus, the cells were treated with 0.05% trypsin (Gibco) for 5 min and proteinase K (TaKaRa) for 2 min, and the total RNA were extracted by RNAiso Plus (TaKaRa) according to the manufacturer’s instructions. The viral RNA was quantitated by RT-qPCR and normalized to GAPDH for cell counting [28].

### Inhibitor inhibition assay

To detect the influence of LDC1267 on replication of CSFV, PK-15 cells were preincubated with increasing concentrations of LDC1267 or DMSO for 30 min at 37°C. Then the cells were infected with CSFV-SM (MOI = 0.01) in the continuous presence of drug. At 48 hpi, relative luciferase activities were detected as described above.

For the investigation of the effect of LDC1267 on the type I IFN signalling, PK-15 cells were preincubated for 30 min with 10 μM LDC1267 or DMSO prior to infection with CSFV-SM (MOI = 0.01) in the continuous presence of drug. Total cellular RNA was extracted at different times and relative IFN-β mRNA levels were measured by RT-qPCR.

### Statistical analysis

Statistical analyses were performed using GraphPad Prism 5 software (GraphPad Software Inc.). Differences between groups were examined for statistical significance using Student’s *t*-test. An unadjusted *P* value of < 0.05 was considered to be significant.

## Results

### Screening of candidate membrane proteins involved in CSFV infection in PK-15 cells

To investigate functional cellular membrane protein(s) engaged in CSFV infection, we first analyzed the documented transcriptomic data of peripheral blood mononuclear cells (PBMCs) before and during 3 days post-infection with different virulent CSFV strains, and selected membrane proteins, which were upregulated by two CSFV strains, as the candidate proteins [29]. Then the results were complemented by another transcriptomic data from pig peripheral blood leukocytes (PBLs) following infection with the CSFV-SM [30]. Finally, twelve candidate proteins were found and then validated by RNA interference (RNAi) screening with rCSFV-Rluc, a *Renilla* luciferase (Rluc) reporter CSFV [24].

**Figure 1.**
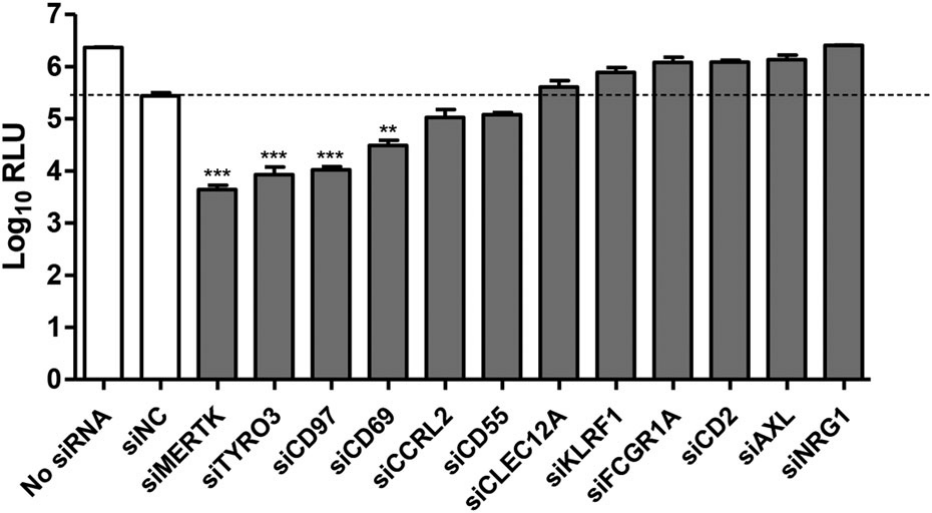
Screening of candidate membrane protein(s) required for CSFV infection. PK-15 cells were transfected with a pool of three siRNAs targeting the 12 candidate proteins or with siNC, and then were infected with rCSFV-Rluc (MOI=0.01). The luciferase activity was measured at 48 hpi. RLU, relative light units. Mean ± standard deviations (SD) of three technical replicates are shown. **, *P* < 0.01; ***, *P* < 0.001.

The results of RNAi screening indicated that silencing of MERTK, TYRO3, CD97, and CD69 significantly reduced luciferase expression in PK-15 cells (Figure 1). We prioritized MERTK for follow-up analysis, since MERTK-knockdown resulted in the strongest reduction of luciferase expression (around 100 folds).

### Verification of the RNAi screening

**Figure 2.**
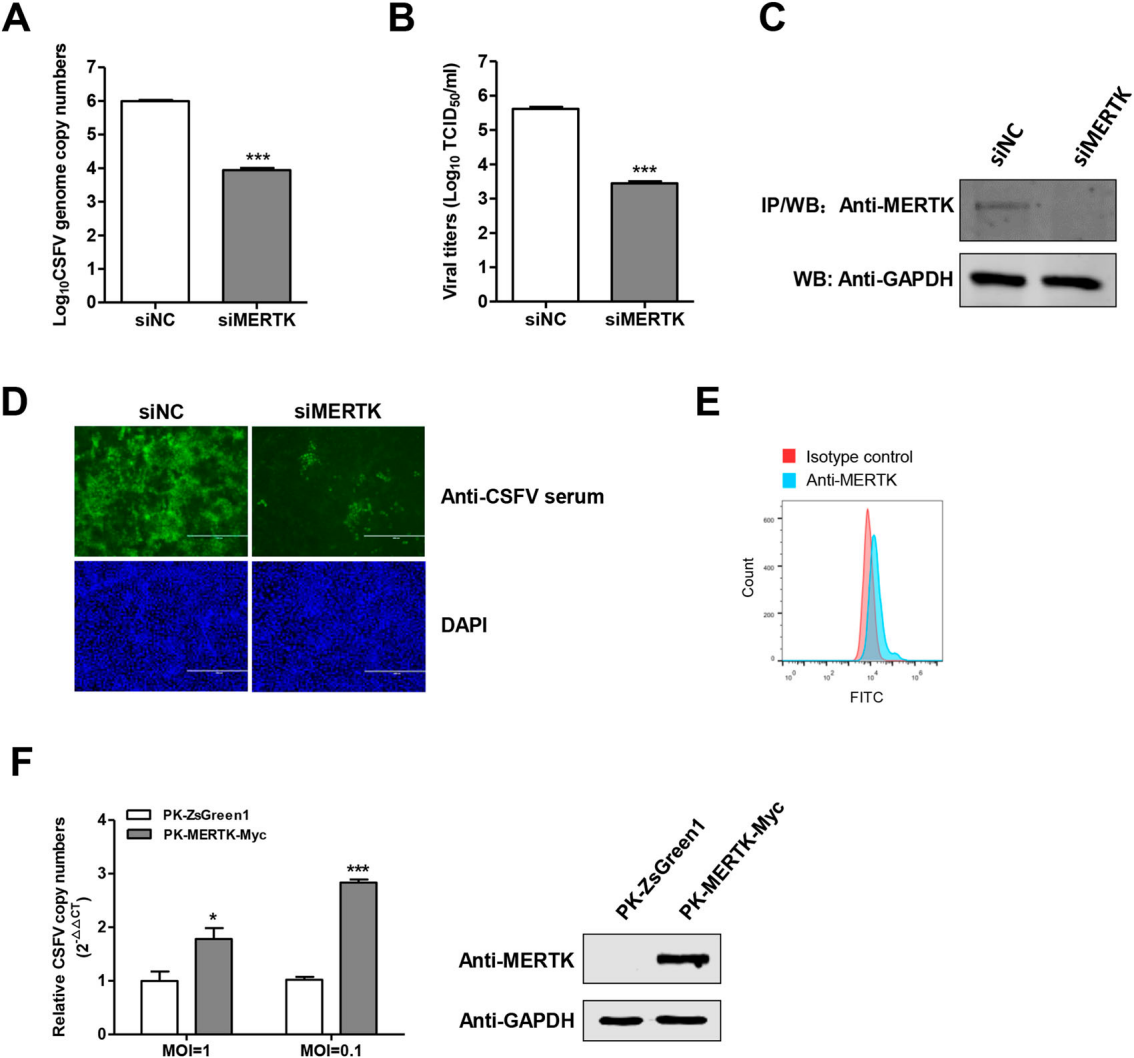
MERTK plays a key role in CSFV infection. (A and B) Silencing of MERTK dramatically reduced CSFV infection. PK-15 cells were treated with siRNAs target to MERTK transcript, and then were infected with CSFV-SM (MOI=0.01) for 48 h, the viral genome copies were quantified (A) and virus titers of supernatants were determined (B) at 48 hpi. (C) Immunoprecipitation assay confirmed that the endogenous MERTK protein expression was decreased in siMERTK treated cells. (D) PK-15 cells were transfected with siMERTK or siNC for 48 h, and were incubated with CSFV-SM (MOI=0.01) for 48 h. Immunofluorescence with pig anti-CSFV sera (green) and DAPI (blue) (E) MERTK expose to the surface of PK-15 cells. The cells were stained with anti-MERTK antibodies or isotype IgG, and then were incubated with secondary antibodies and followed by flow cytometric analysis of 10,000 cells per sample. (F) Overexpression of MERTK enhances CSFV infection. PK-MERTK-Myc or PK-ZsGreen1 cells were infected with CSFV-SM (MOI=0.01), the CSFV genomic replication was determined at 24 hpi. Insets display overexpression of MERTK-Myc. Means ± standard deviations (SD) of three technical replicates are shown. *, *P* < 0.05; **, *P* < 0.01; ***, *P* < 0.001.

To further validate the role of MERTK in CSFV infection, PK-15 cells were infected with the CSFV-SM after MERTK silencing. The results showed that the knockdown of MERTK dramatically decreased the intracellular viral genome replication and the progeny viruses in the supernatants at 48 h post-infection (hpi) (Figure 2A and B). The knockdown efficiency of endogenous MERTK protein was examined using co-immunoprecipitation (Co-IP) (Figure 2C), since the endogenous MERTK protein was hardly detectable by Western blotting. In addition, we assessed the CSFV replication in MERTK-knockdown cells using immunofluorescence at 48 hpi, and found that the fluorescence of CSFV in MERTK-knockdown cells was significantly decreased compared with negative control siRNA (siNC)-treated cells (Figure 2D).

Next, we tested the cell surface expression of MERTK on PK-15 cells. Flow cytometry analysis revealed that MERTK was exposed to the cell surface, albeit at low levels (Figure 2E). Furthermore, using a lentivirus delivered MERTK-overexpressing cell line (PK-MERTK-Myc), we showed that overexpression of MERTK enhanced the replication of CSFV of different MOIs about 2–3 folds at 24 hpi (Figure 2F). Collectively, these data further support the key role of MERTK in CSFV infection.

### Anti-MERTK antibodies or soluble MERTK ectodomain reduce CSFV infection

**Figure 3.**
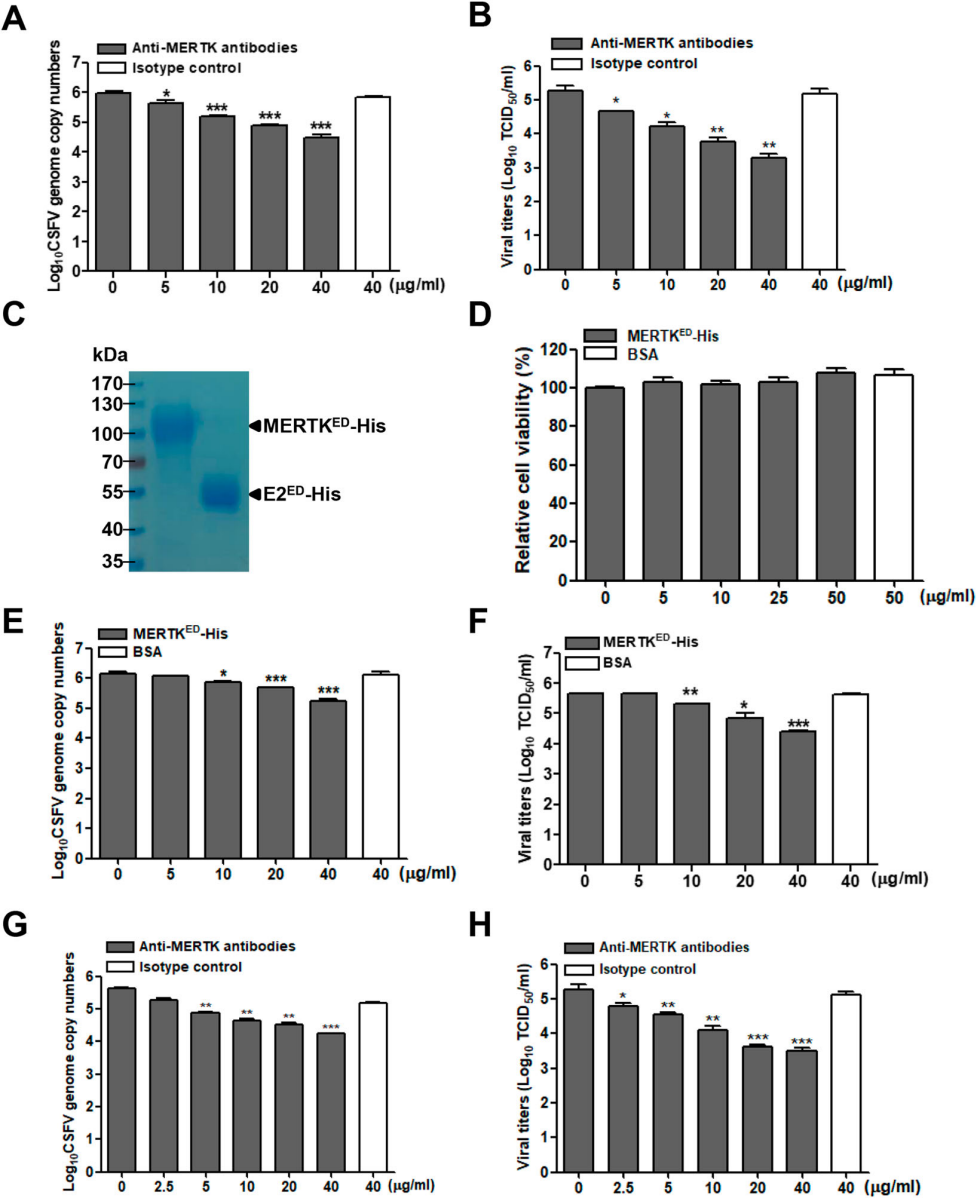
Anti-MERTK antibodies or soluble MERTK ectodomain blocks CSFV infection in a dose-dependent manner. (A and B) Antibodies against MERTK reduce CSFV infection. PK-15 cells were pretreated with antibodies against MERTK or isotype IgG at indicated concentrations for 30 min, and then infected with CSFV-SM (MOI=0.01) in the continuous presence of the antibodies. The viral genome copies (A) and progeny viral titers (B) were measured at 48 hpi. (C) Identification of purified MERTK^ED^-His and E2^ED^-His proteins stained with Coomassie blue. (D) MERTK^ED^-His has no effects on the cell viabilities. (E and F) Soluble MERTK^ED^-His reduces CSFV infection. CSFV-SM (MOI=0.01) was incubated with the indicated concentrations of soluble MERTK^ED^-His or BSA for 30 min at room temperature, and then infected with PK-15 cells. The viral genome copies (E) and progeny viral titers (F) were measured at 48 hpi. (G and H) Anti-MERTK antibodies reduce CSFV-HLJ infection in a dose-dependent manner. PK-15 cells were infected with CSFV-HLJ (MOI=0.01) after incubation with anti-MERTK antibodies for 30 min. The viral genome copies (G) and progeny viral titers (H) were examined at 48 hpi. Means ± standard deviations (SD) of three technical replicates are shown. **, *P* < 0.01; ***, *P* < 0.001.

To further assess the contributions of MERTK to CSFV infection. PK-15 cells were pre-treated with different concentrations of anti-MERTK antibodies for 30 min at 37°C, and then infected with CSFV-SM. We observed that anti-MERTK antibodies reduced the viral genome copies (Figure 3A) and progeny viral titers (Figure 3B) in a dose-dependent manner. Additionally, we expressed and purified the soluble MERTK ectodomain (MERTK^ED^-His) for blocking assay (Figure 3C), and MERTK^ED^-His has no cytotoxicity on cell viability within 50 μg/mL (Figure 3D). CSFV-SM was pre-treated with MERTK^ED^-His or BSA for 30 min at room temperature, and then the mixtures were inoculated into PK-15 cells for 48 h. The results demonstrated that the viral genome copies and progeny viral titers were also reduced by soluble MERTK^ED^-His in a dose-dependent manner (Figure 3E and F), indicating that MERTK is involved in CSFV entry.

Additionally, we addressed whether MERTK is involved in the infection of genotype 2 CSFV-HLJ in PK-15 cells. The blocking assay results showed that anti-MERTK antibodies also reduced the CSFV-HLJ genome copies and progeny viral titers in a dose-dependent manner (Figure 3G and H). Together, these data suggest that MERTK plays an important role in CSFV infection.

### MERTK interacts with the CSFV E2 protein

**Figure 4.**
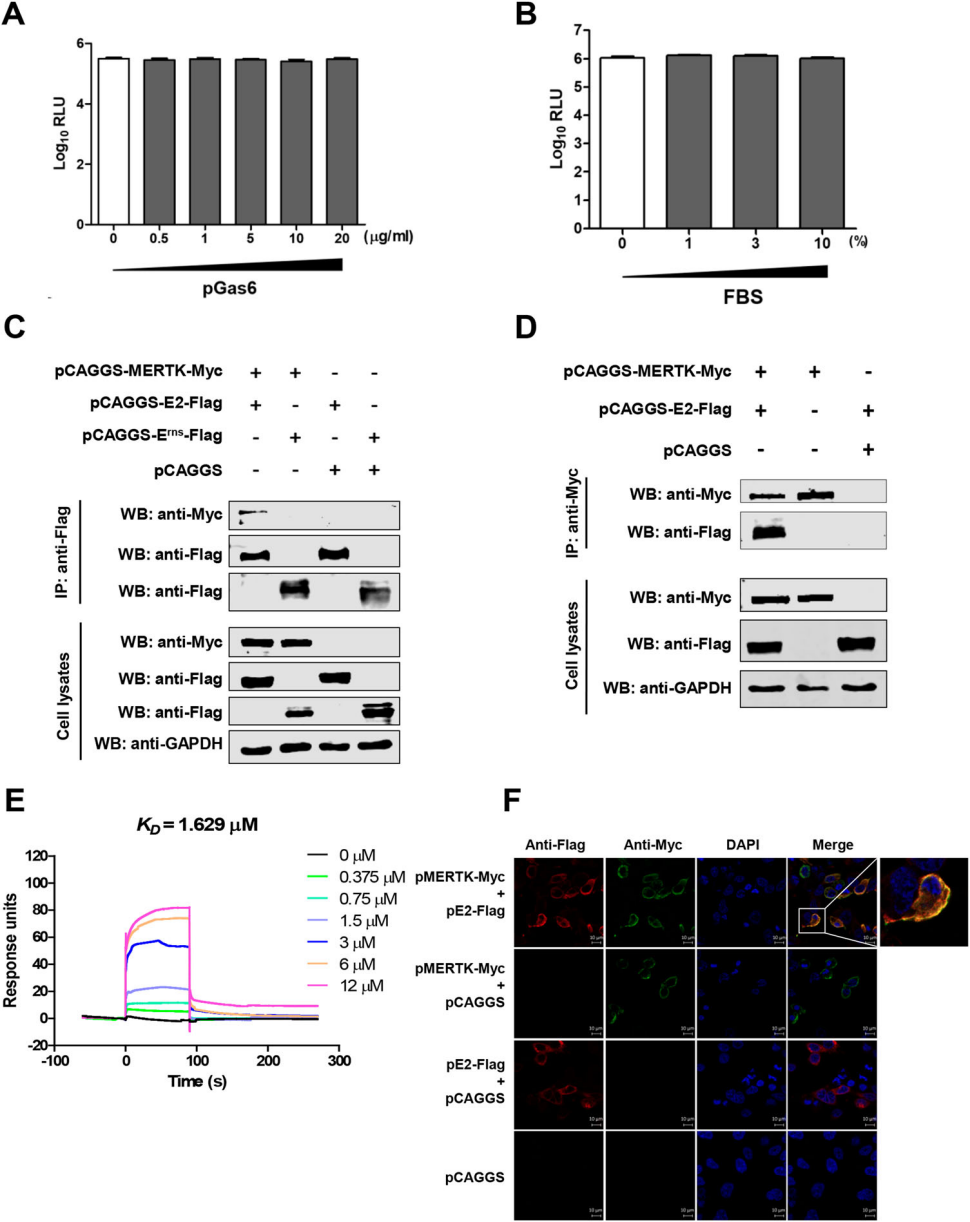
MERTK binds to the CSFV E2 protein. (A and B) Gas6 and FBS have no effect in CSFV infection. Serum-starved PK-15 cells were incubated with CSFV-Rluc (MOI=0.01) in serum-free medium containing the indicated concentrations of pGas6 (A) or FBS (B). After 2 h, medium was replaced by medium supplemented with 4% FBS, and relative light units (RLU) were determined at 48 hpi. (C and D) MERTK interacts with the CSFV E2 protein. HEK293 T cells were co-transfected with pMERTK-Myc and pE2-Flag or pE^rns^-Flag. At 48 hpt, the cells lysates were collected, and then subjected to Co-IP analysis using anti-Flag MAb (C) or anti-Myc MAb (D). (E) Affinity analysis of MERTK^ED^-His and E2^ED^-His. Surface plasmon resonance (SPR) assay characterizes the specific binding between the purified MERTK^ED^-His and E2^ED^-His proteins. (F) MERTK colocalizes with the E2 protein. The expression plasmids pMERTK-Myc and pE2-Flag were co-transfected into HEK293 T cells and subjected to confocal assay. Scale bar, 10 μm.

Several viruses have been reported to bind to the TAM receptor proteins either by indirect bridging ligands or by direct interaction with viral proteins [15]. Growth-arrest-specific 6 (Gas6) and protein S (ProS) are the natural ligands for the TAM receptors [31–33]. ProS is present approximately of 300 nM in fetal bovine serum (FBS) [34, 35], which enhances the infection of many flaviviruses and the transduction of lentiviral vectors pseudotyped with various viral proteins [16, 21, 35]. However, our studies showed that CSFV infection is independent of porcine Gas6 (pGas6) or FBS (Figure 4A and B), indicating that both ligands may not be involved in CSFV infection. Therefore, we reasoned that MERTK protein binds to CSFV glycoproteins. To this end, Co-IP was performed, and the results showed that E2, but not E^rns^, interacts with MERTK (Figure 4C and D). To further confirm the interaction between MERTK and the CSFV E2 protein, we prepared the E2 ectodomain (E2^ED^-His) for surface plasmon resonance (SPR) analysis (Figure 3C). The results showed that MERTK^ED^-His bound to E2^ED^-His in a dose-dependent manner (Figure 4E). The equilibrium dissociation constant (*K_D_*) value between MERTK^ED^-His and E2^ED^-His was 1.629 μM. Correspondingly, the colocalization of MERTK and E2 was confirmed in HEK293 T cells, which transiently overexpress the two proteins (Figure 4F). Together, these data indicate that MERTK interacts with the CSFV E2 protein.

### MERTK facilitates CSFV entry

**Figure 5.**
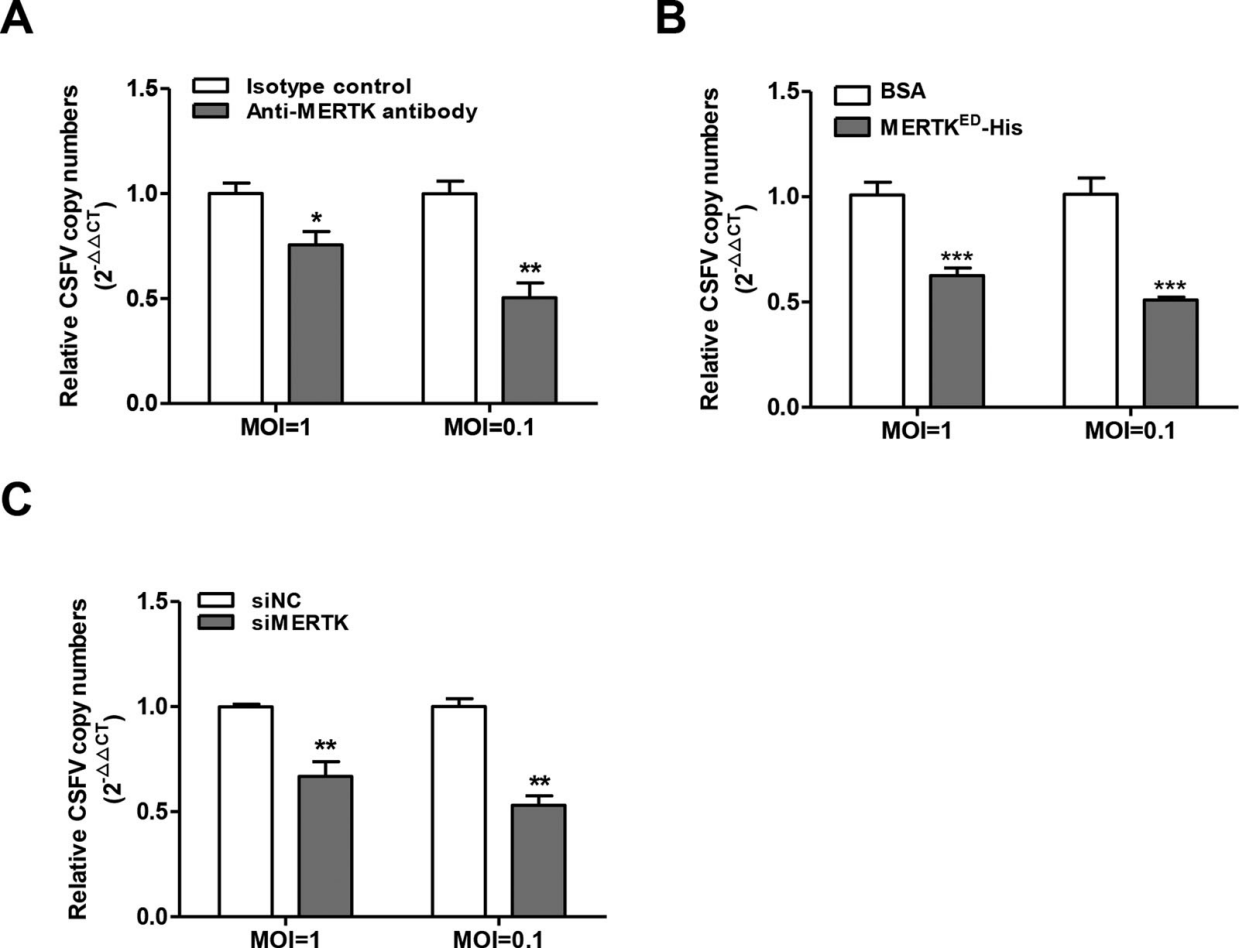
MERTK promotes CSFV entry. (A) Anti-MERTK antibodies decrease CSFV entry. PK-15 cells were incubated with anti-MERTK antibodies or isotype IgG and were incubated with CSFV-SM (MOI=0.1 or 1) for 2 h in the continuous presence of the antibodies at 37°C for virus entry. Then the cells were subjected to analyze CSFV entry. (B) Soluble MERTK^ED^-His reduces CSFV entry. CSFV-SM (MOI=0.1 or 1) were preincubated with soluble MERTK^ED^-His or BSA for 30 min at room temperature then used to infect PK-15 cells. The infected cells were incubated at 37°C for 2 h. RT-qPCR was performed to determine CSFV genome copies relative to GAPDH. (C) Downregulation of MERTK reduces CSFV entry. PK-15 cells were transfected with MERTK targeting siRNA or siNC and then were analyzed for CSFV entry. Means ± standard deviations (SD) of three technical replicates are shown. *, *P* < 0.05; **, *P* < 0.01; ***, *P* < 0.001.

MERTK is known to mediate the phagocytosis of apoptotic cells, and recent studies have demonstrated that TAM receptors mediate the entry of several viruses (ZIKV, DENV, ZEBOV, and others) [36–38]. Moreover, our results demonstrated that CSFV infection can be reduced by anti-MERTK antibodies or soluble MERTK^ED^-His. Therefore, these observations inspired us to identify whether MERTK plays a role in CSFV entry. PK-15 cells were incubated with CSFV at 37°C for 2 h after relevant treatment with anti-MERTK antibodies or soluble MERTK^ED^-His, and then treated with trypsin and proteinase K to remove the bound virions on the cell surface. Viral RNA was quantified by RT-qPCR and normalized to GAPDH for cell counting. The results showed that both anti-MERTK antibodies and soluble MERTK^ED^-His decreased CSFV entry into PK-15 cells (Figure 5A and B). Furthermore, we demonstrated that MERTK knockdown also reduced CSFV entry (Figure 5C). In summary, these results demonstrate that MERTK facilitates the entry of CSFV into PK-15 cells.

### MERTK antagonizes type I IFN signalling pathway in CSFV infection

**Figure 6.**
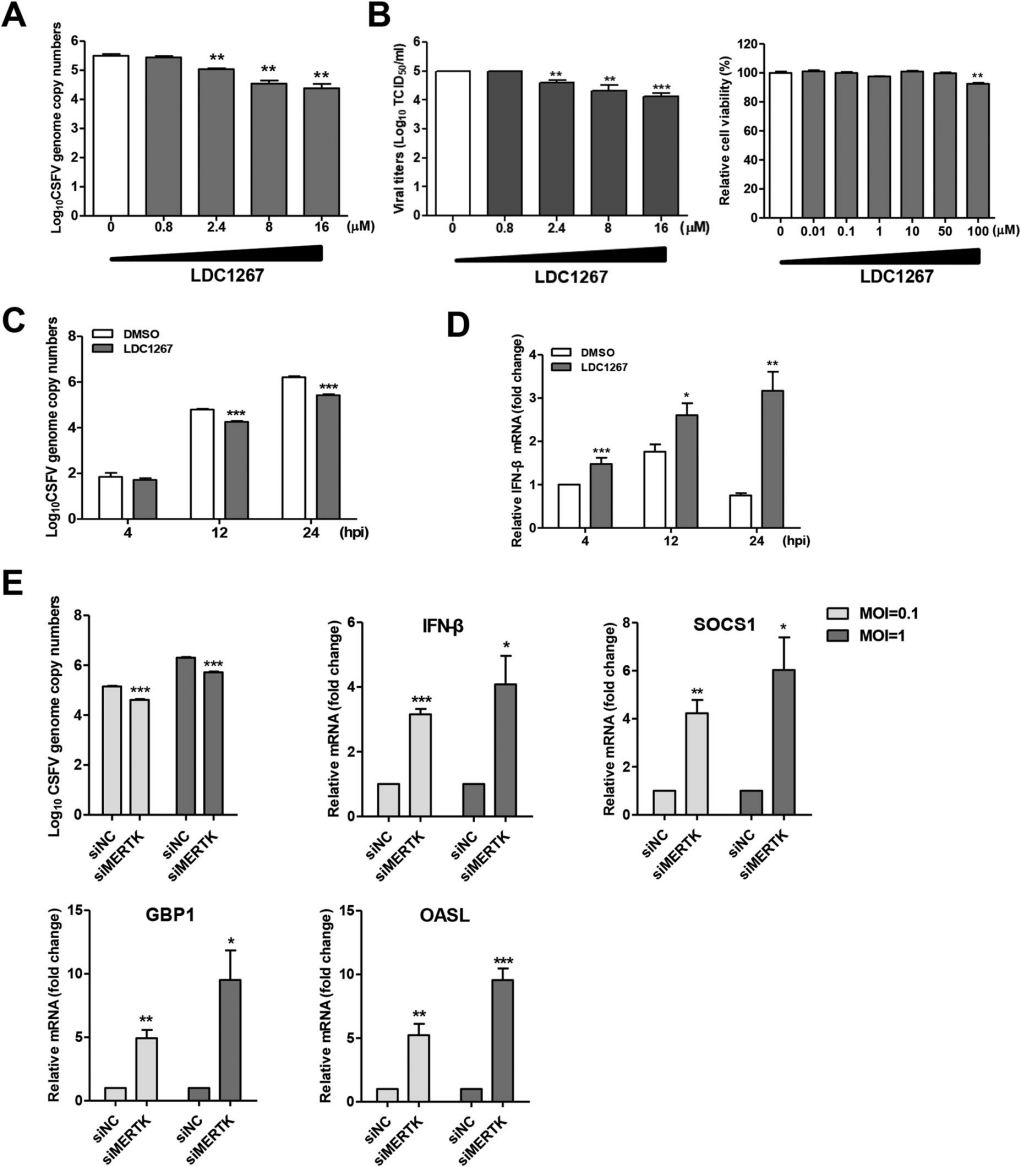
MERTK dampens innate immune response in CSFV infection of PK-15 cells. (A and B) LDC1267 reduced CSFV infection in a dose-dependent manner. PK-15 cells were pre-incubated with increasing concentrations of LDC1267 or DMSO for 30 min and were infected with CSFV-SM (MOI=0.01) in presence of the drug. At 48 hpi, the CSFV-SM genome copies (A) and progeny viral titers (B) were measured. The relative cell viability was determined by using CCK-8. (C and D) PK-15 cells were pre-incubated with LDC1267 (10 μM) or DMSO for 30 min, and cells were challenged with CSFV-SM (MOI=0.01) in the presence of the drug. Total cellular RNA was extracted at indicated time point, and relative viral RNA copies (C) and IFN-β mRNA level (D) were determined by RT-qPCR. (E) PK-15 cells transfected with siRNA targeting MERTK or negative control were infected with CSFV-SM at different MOIs (1 or 0.1). Total cellular RNA was extracted at 24 h, and relative viral RNA copies and IFN-β, SOCS1, GBP1, and OASL mRNA levels were determined by RT-qPCR. Means ± standard deviations (SD) of three technical replicates are shown. *, *P* < 0.05; **, *P* < 0.01; ***, *P* < 0.001.

It has been reported that the activation of TAM receptors inhibits innate immune response [28]. Some enveloped viruses utilize TAM receptors to inhibit type I IFN signalling, thereby promoting viral infection [16, 21]. To determine whether MERTK kinase activity enhances CSFV infection, we chose LDC1267, a selective inhibitor of TAM kinase activity [39], to perform the inhibition assay and found that CSFV replication was reduced in a dose-dependent manner (Figure 6A and B). Moreover, LDC1267 treatment reduced CSFV replication and upregulated IFN-β mRNA levels at a later time point (Figure 6C and D). To further verify that MERTK antagonizes the host innate immune response to CSFV, we quantified the mRNA levels of IFN-β and suppressor of cytokine signalling protein 1 (SOCS1) in MERTK-knockdown cells infected with CSFV at 24 hpi. Our results showed that silencing of MERTK resulted in increased mRNA levels of IFN-β and SOCS1, as well as of GBP1 and OASL (Figure 6E), which have been identified as potent anti-CSFV interferon-stimulated genes (ISGs) [27,40]. Taken together, these findings suggest that MERTK facilitates CSFV entry and enhances viral replication.

### MERTK also plays an important role in BVDV infection

**Figure 7.**
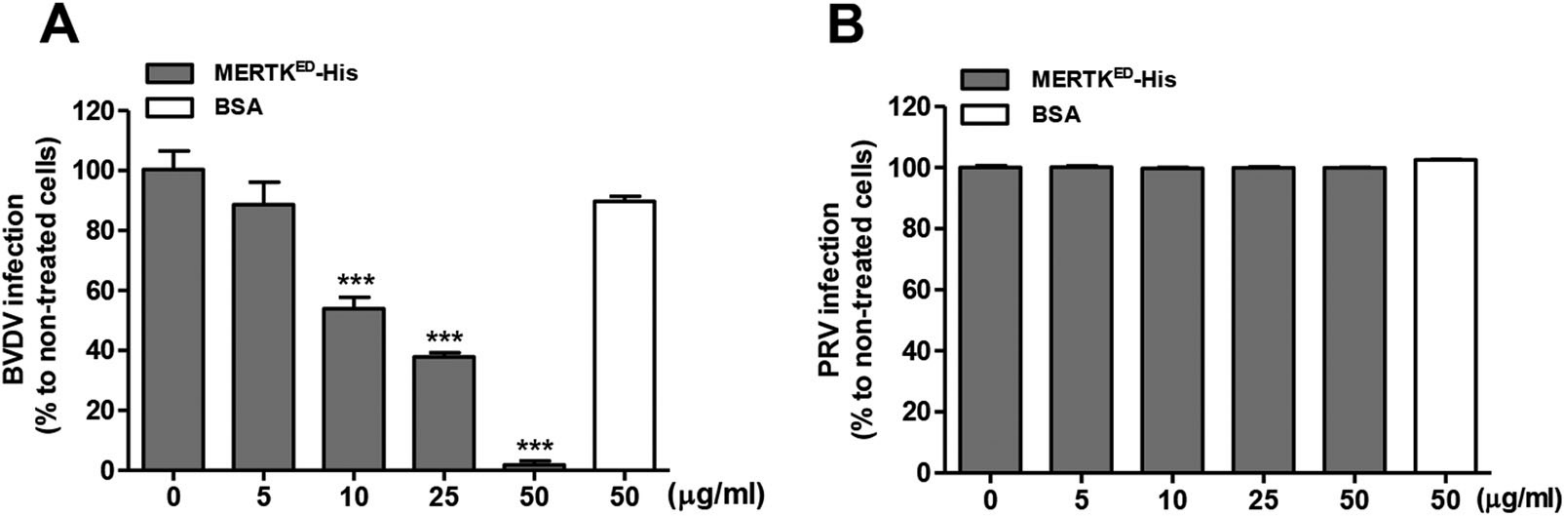
Soluble MERTK ectodomain reduces BVDV infection in a dose-dependent manner. (A) BVDV-C24 V (MOI=0.01) was incubated with the indicated concentrations of soluble MERTK^ED^-His, or BSA for 30 min at room temperature, and then was incubated with MDBK cells. Total RNA of the cells was extracted at 24 hpi, and relative viral RNA was detected by RT-qPCR. (B) The PRV-TJ (MOI=0.01) was preincubated with various concentrations of soluble MERTK^ED^-His or BSA for 30 min and inoculated into PK-15 cells. Relative PRV genome was determined by qPCR. Mean ± standard deviations (SD) of three technical replicates are shown. *, *P* < 0.05; **, *P* < 0.01; ***, *P* < 0.001.

BVDV, along with CSFV, belongs to the *Pestivirus* genus. Hence, we wondered whether MERTK also plays a functional role in BVDV infection. BVDV-C24 V and PRV-TJ were tested in the blocking assays. PRV together with herpes simplex virus type 1 (HSV-1), of which infection is independent of TAM receptors [22], belong to the family *Herpesviridae*, indicating that MERTK is not required for PRV infection. BVDV-C24 V or PRV-TJ particles were pretreated with different concentrations of soluble MERTK^ED^-His for 30 min at room temperature, and then were incubated with MDBK or PK-15 cells for 24 h, respectively. As expected, the results demonstrated that soluble MERTK^ED^-His decreased BVDV infection in a dose-dependent manner (Figure 7A), but displayed no effects on PRV infection (Figure 7B). Overall, our data suggest that MERTK is an important host factor for pestiviral infection.

## Discussion

To date, host membrane proteins involved in the entry of CSFV are not well characterized, and understanding these interactions would establish foundations for future therapeutics and immunization strategies. In this study, we have shown that MERTK is a novel host factor that binds to the E2 protein of CSFV to promote CSFV entry and immuno-evasion in PK-15 cells, giving an insight into the events of CSFV life cycle.

Recent studies have shown that TYRO3 and AXL are involved in flaviviruses infection. For example, ectopic expression of TYRO3 and AXL enhances infection of all DENV serotypes, as well as other related viruses such as WNV and YFV [13,14,16]. Additionally, AXL acts as a ZIKV entry receptor in human skin cells, neural stem cells and human glial cells [19–21]. However, the involvement of MERTK, another member of the TAM receptors, in viral infection remains unknown. Our study first demonstrated that MERTK plays an important role in pestiviral infection, providing a new insight on the role of TAM receptors in flaviviruses infection.

Viruses have evolved various strategies to hijack TAM receptors to facilitate their infections [15]. Generally, there are two mechanisms used by different viruses to bind the TAM receptors. One is that some viruses indirectly interact with TAM receptors via the bridging molecular (Gas6 or ProS), such as DENV and ZIKV [16,21]. Another is that viruses directly bind to the TAM receptors. Specifically, the VP1 protein of SV40 could structurally mimic Gas6 to directly interact with AXL to enhance viral infection [23]. Moreover, TYRO3 binds to Lassa virus pseudotype particles, indicating that TYRO3 directly binds to viral particles [41]. In the present study, we demonstrated that MERTK binds to the CSFV E2 protein and promotes virus entry, adding the list of various mechanisms of TAM receptors used by different viruses.

TAM family kinase receptors are important negative regulators of the type I IFN signalling pathway. It has been demonstrated that stimulation of the type I IFN signalling upregulates TAM proteins, which in turn leads to the production of SOCS1 and SOCS3 to suppress the type I IFN signalling [34,42]. This self-regulating mechanism is a key component of the innate immune system [42]. Many viruses hijack the TAM receptors to facilitate viral infection of target cells. For example, flaviviruses and pseudotyped retroviruses activate TAM receptors kinase activities to suppress type I IFN expression to facilitate viral replication in dendritic cells [14]. Moreover, ZIKV upregulates SOCS1 to antagonize the type I IFN signalling by activating kinase activities of TAM receptors in human glial cells [22]. In this study, we showed that MERTK antagonizes the type I IFN signalling pathway after CSFV entry to PK-15 cells, enriching the understanding of the TAM receptors involvement in innate immunity.

TAM receptors are highly conserved among different animal species and are widely distributed in various cell types [43]. The interaction between MERTK and E2 might be associated with some clinical features of CSF. Firstly, MERTK has been detected in PBMCs, bone marrow mononuclear cells, monocytes, and macrophages [32,44], and is also enriched in spleen, lymph nodes, and thymus [45], which is possibly consistent with the host tropism of CSFV. Moreover, MERTK has also been detected in spermatogonia, leydig, and sertoli cells of the testes [46,47].

To investigate whether anti-MERTK antibodies or soluble MERTK^ED^-His blocking affect the interferon response in PK-15 cells, we treated PK-15 cells with anti-MERTK antibodies or soluble MERTK^ED^-His for 4 h, and then quantified the mRNA levels of IFN-β. No significant difference was observed between the treatment and the control cells (data not shown), indicating that the role of anti-MERTK antibodies and soluble MERTK^ED^-His in CSFV infection might depend on the blockage of the MERTK-E2 interaction to decrease the entry of CSFV.

In summary, we have demonstrated, for the first time, that the functional role of MERTK in pestiviral infections, gaining further insights into the mechanism of CSFV infection and providing a potential target of therapies to augment the response to pestiviral infections.
